# Structures, Sources, Identification/Quantification Methods, Health Benefits, Bioaccessibility, and Products of Isorhamnetin Glycosides as Phytonutrients

**DOI:** 10.3390/nu15081947

**Published:** 2023-04-18

**Authors:** Hong Wang, Lijia Chen, Binrui Yang, Jun Du, Liang Chen, Yiming Li, Fujiang Guo

**Affiliations:** 1 School of Pharmacy, Shanghai University of Traditional Chinese Medicine, Shanghai 201203, China; 2 Nutrition Science, Amway (Shanghai) Innovation & Science Co., Ltd., Shanghai 201203, China

**Keywords:** isorhamnetin glycosides, phytonutrients, health-promoting effects, sources

## Abstract

In recent years, people have tended to consume phytonutrients and nutrients in their daily diets. Isorhamnetin glycosides (IGs) are an essential class of flavonoids derived from dietary and medicinal plants such as *Opuntia ficus-indica*, *Hippophae rhamnoides*, and *Ginkgo biloba*. This review summarizes the structures, sources, quantitative and qualitative analysis technologies, health benefits, bioaccessibility, and marketed products of IGs. Routine and innovative assay methods, such as IR, TLC, NMR, UV, MS, HPLC, UPLC, and HSCCC, have been widely used for the characterization and quantification of IGs. All of the therapeutic effects of IGs discovered to date are collected and discussed in this study, with an emphasis on the relevant mechanisms of their health-promoting effects. IGs exhibit diverse biological activities against cancer, diabetes, hepatic diseases, obesity, and thrombosis. They exert therapeutic effects through multiple networks of underlying molecular signaling pathways. Owing to these benefits, IGs could be utilized to make foods and functional foods. IGs exhibit higher bioaccessibility and plasma concentrations and longer average residence time in blood than aglycones. Overall, IGs as phytonutrients are very promising and have excellent application potential.

## 1. Introduction

Phytonutrients are chemical compounds that are only present in natural plants and are beneficial to the human body [1]. They are widely used in food and nutraceuticals due to their health-promoting benefits [2]. Flavonoids are a class of polyphenolic compound distributed in many fruits, vegetables, and plants [3]. The six major subclasses of flavonoids, which include flavones (e.g., luteolin), flavonols (quercetin), flavanones (hesperidin), catechins or flavanols (epicatechin), anthocyanidins (cyanidin), and isoflavones (daidzein), have been reported to represent various families of phytonutrients [4]. Accumulating evidence based on observational and clinical studies shows that a plant-based dietary pattern rich in fruits, vegetables, and whole grains has a clear effect on the prevention of various chronic diseases [5], and people also tend to consume dietary flavonoids from fruits and vegetables. Flavonoids are widely found in food, and most of them exist in their glycosidic forms [6,7].

Isorhamnetin glycosides (IGs), as natural flavonol compounds, are primarily extracted from various plant-based foods or medicinal plants such as *Opuntia ficus-indica*, *Hippophae rhamnoides*, and *Ginkgo biloba* [8,9,10]. IGs are biologically important flavonols with proven beneficial properties that give them medicinal value [11,12]. They possess diverse biological and pharmacological properties, such as antioxidant, anti-inflammatory, anti-cancer, antidiabetic, anti-obesity, and hepatoprotective properties [13,14,15,16,17]. Due to their beneficial biological activities, IGs have been considered a significant potential class of phytonutrients, and an increasing number of products containing IGs are circulating on the market in many countries, including the United States, Canada, Mexico, China, India, and some European countries [18,19].

Here, for the first time, a review of all studies that describe the biological activity of IGs is presented, with particular emphasis on molecular signaling pathways and mechanistic explanations for their health-promoting potential. This review also introduces the structure of IGs and the primary sources of IGs. Moreover, current methods for the analysis and quantification of IGs are summarized. Furthermore, this paper also focuses on the main bioaccessibility of IGs. Overall, this article strongly supports the use of IGs as phytonutrients.

## 2. Structure of IGs

**Figure 1:**
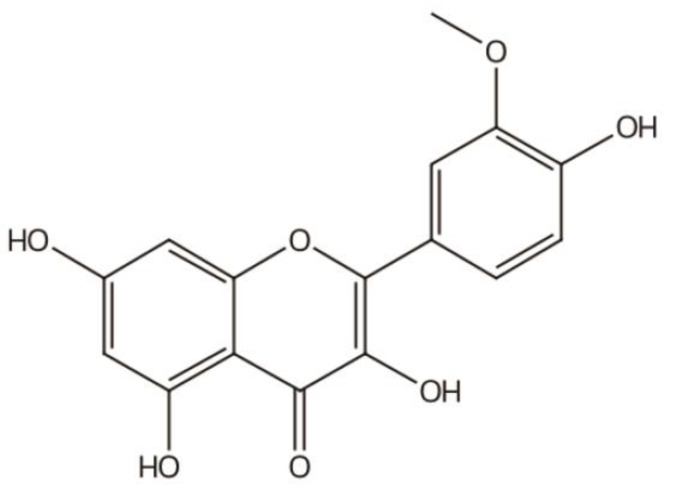
Basic parent nucleus of isorhamnetin glycosides (IGs).

IGs are a type of glycosylated flavonol composed of an isorhamnetin skeleton and sugar groups. Their aglycone isorhamnetin, i.e., 3,4′,5,7-tetrahydroxy-3′-methoxyflavone, is an *O*-methylated flavonol (Figure 1). Generally, d-glucose, d-galactose, l-rhamnose, d-xylose, l-arabinose, sophorose, and rutinose are the most common sugar groups of IGs. They are linked to the aglycone by an *O*-glycosidic bond. According to the number of sugar groups, IGs are classified as mono-, di-, tri-, or tetra-glycosides. Position substitutions mostly happen at C-3 and C-7, for example, isorhamnentin-3-*O*-β-d-glucoside (**4**) and isorhamnetin-3-*O*-β-d-glucoside-7-*O*-α-l-rhamnoside (**20**) from *Hippophae rhamnoids* [20]; isorhamnetin-3-*O*-α-l-rhamnoside (**3**) from Laportea bulbifera Wedd. [21]; and isorhamnetin-7-*O*-β-d-glucoside (**1**) and isorhamnetin-7-*O*-α-l-rhamnoside (**2**) from Nitraria tangutorum Bolor [22]. Of course, sometimes, substitution occurs at C-4′, for instance, isorhamnetin-4′-*O*-β-d glucoside (**9**) from Allium cepa L. [23]; isorhamnetin-3,4′-*O*-β-d-diglucoside (**17**) from Allium ascalonicum [24]; isorhamnetin-3-*O*-β-d -glucoside-4′-*O*-β-d-xyloside (**21**) [25]; and isorhammetin-3-*O*-α-l-rhamnoside-(1→6)-β-d-glucoside-4′-*O*-β-d-glucoside (**35**) [26]. In addition, some sugar group derivatives, such as isorhamnetin-3-*O*-[2‴-*O*-acetyl−β-d-xyloside-(1→6)-β-d-glucoside] (**10**) [27] and isorhamnetin-3-*O*-β-d (6-acetyl-glucoside) (**7**) [28], have also been obtained.

**Figure 2:**
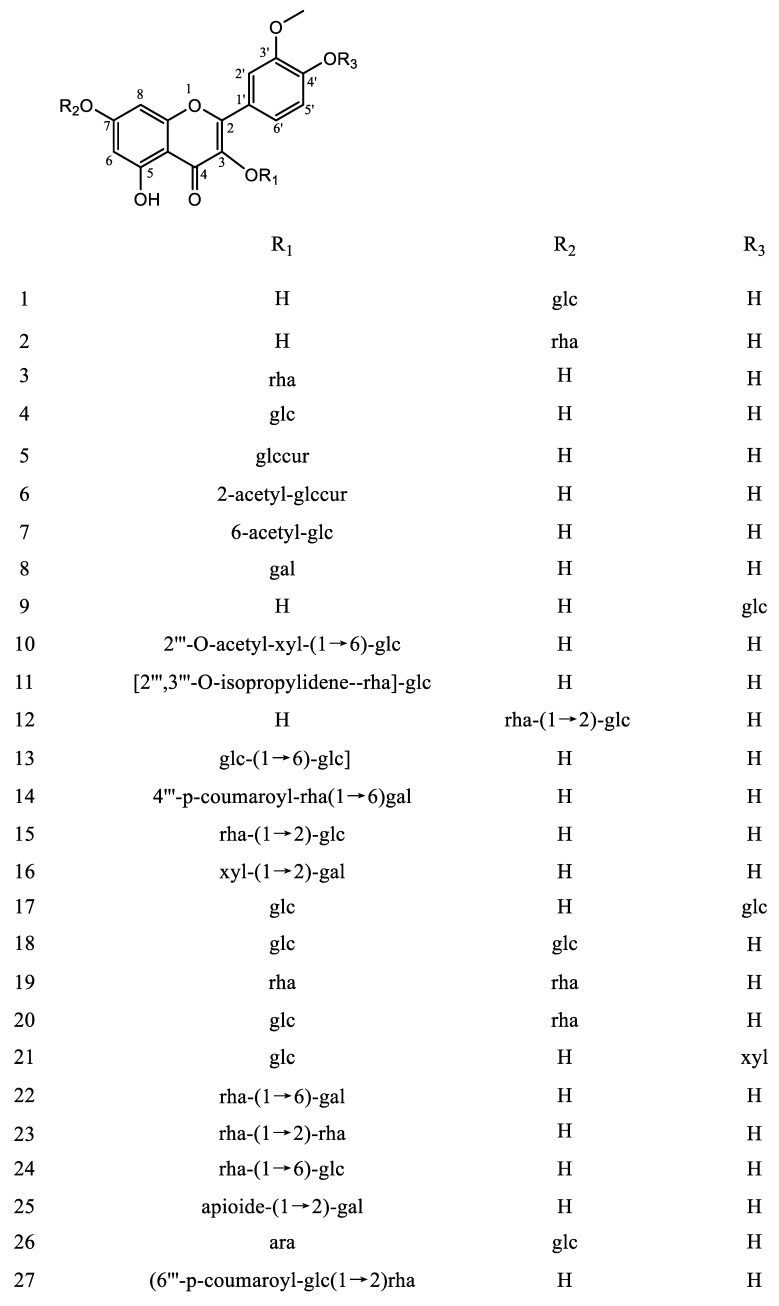

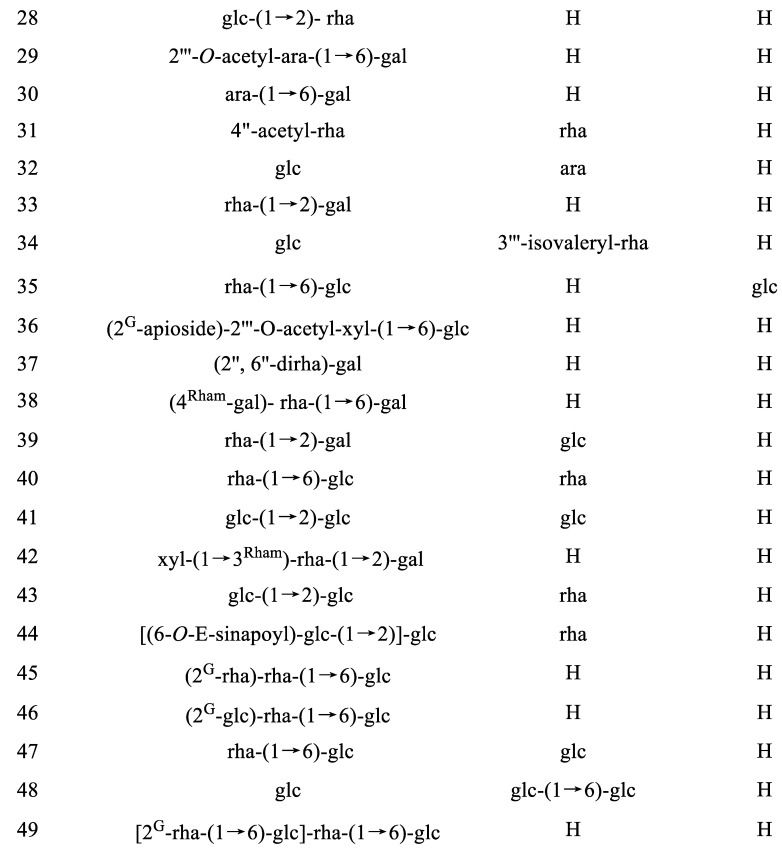
Chemical structures of IGs (compounds **1**–**49**). Monoglycosides (**1–9**), diglycosides (**10–34**), triglycosides (**35–48**), and tetraglycosides (**49**). Abbreviations: Glc: D-glucose, Rha: L-rhamnose, Glccur: D-glucuronic, Gal: D-galactose, Xyl: D-xylose, Ara: L-arabinose. Abbreviations: Glc: d-glucose, Rha: l-rhamnose, Glccur: d-glucuronic, Gal: d-galactose, Xyl: d-xylose, Ara: l-arabinose.

In the present review, we systematically summarize the 49 compounds of IGs reported thus far (Table 1 and Figure 2).

## 3. Sources of IGs

**Figure 3:**
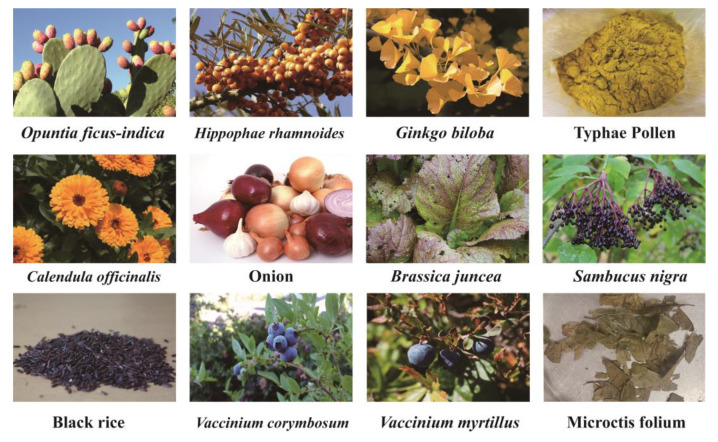
Plants with IG content.

IGs as nutritional supplements can be obtained from some foods and medicinal plants. Commonly consumed foods containing IGs include *Hippophae rhamnoides*, *Opuntia ficus-indica*, *Vaccinium corymbosum*, *Vaccinium myrtillus*, *Brassica juncea*, rice, and onions. The main medicinal sources of Igs are *Ginkgo biloba*, pollen Typhae, Microctis folium, *Sambucus nigra*, and *Calendula officinalis* (Figure 3).

### 3.1. Opuntia ficus-indica

*Opuntia ficus-indica*, otherwise known as the prickly pear or nopal cactus, is a multipurpose crop that grows wild in the arid and semi-arid regions of the world [70]. It is used not only in the diet to provide food and feed, but also for healthcare due to its antioxidant, anti-inflammatory, and anxiolytic properties [71,72].

IGs have already been described to be the most abundant flavonoid in *Opuntia ficus-indica* [8, 73–74] and in different *Opuntia* species [73]. Variable amounts of IG distributed in the cladode, pulp, and peel of the *Tunisian Opuntia ficus-indica* have been investigated [74]. Isorhamnetin-3-*O*-rutinoside (**24**) was found at very high and significant levels in the cladodes (703.33 ± 28.45 mg/100 g, DW (dry weight)), pulps (271.39 ± 25.59 mg/100 g, DW), and peels (254.51 ± 31.03 mg/100 g, DW). Moreover, isorhamnetin-3-*O*-glucoside (**4**) was also found in the cladodes (149.71 ± 10.13 mg/100 g, DW), pulps (184.14 ± 14.91 mg/100 g, DW) and peels (223.66 ± 14.44 mg/100 g, DW).

### 3.2. Hippophae rhamnoides

*Hippophae rhamnoides* (also named sea buckthorn) [20] constitutes a rich source of IGs [10]. Its berries have been categorized as a “medicine food homology” fruit by China’s National Health Commission for both nutritional and medicinal purposes [19]. *Hippophae rhamnoides* has a wide range of positive biological, physiological, and medicinal effects, such as antioxidative, anti-inflammatory, antidiabetic, anticarcinogenic, hepatoprotective, and dermatological effects [75].

IGs have been found in all parts of the sea buckthorn plant, including the berries, leaves, and seeds [76]. An investigation of six cultivated *Hippophae rhamnoides* varieties revealed that the berries contained an average of 917 mg/100 g DW of flavonol glycosides [77], whereas the content of flavonol glycosides in leaves was higher than that in berries, with an average of 1118 mg/100 g DW. Isorhamnetin-3-hexoside (75.0~406.1 mg/100 g, DW), isorhamnetin-3-rhamnosylglucoside (**24**) (52.5~190.0 mg/100 g DW), isorhamnetin-3-neohesperidoside (**15**) (110.1~323.8 mg/100 g, DW), and free isorhamnetin were predominant in the berries. Isorhamnetin-3-rhamnoside (**3**) (41.8~159.1 mg/100 g, DW), isorhamnetin-3-glucoside-7-rhamnoside (**20**) (67.6~129.3 mg/100 g, DW), isorhamnetin-3-rhamnosylglucoside (**24**) (66.7~253.0 mg/100 g, DW), isorhamnetin-3-neohesperidoside (**15**) (60.6~172.1 mg/100 g, DW), and isorhamnetin-3-rutinoside-7-glucoside (**47**) (36.0~117.3 mg/100 g, DW) were predominant in the leaves. Another study determined the content of IG from the berries of different cultivars of sea buckthorn. It was found that isorhamnetin derivatives represented over 65% of the total flavonols in sea buckthorn berries [78]. Isorhamnetin-3-*O*-rutinoside (**24**) had the highest content, in the range of 96.4~228 mg/100 g dry matter (DW). The study also confirmed that high concentrations of isorhamnetin-3-*O*-glucoside (**4**) (62.0~217.0 mg/100 g, DW) and isorhamnetin-3-*O*-glucoside-7-*O*-rhamnoside (**20**) (37.8~90.8 mg/100 g, DW) were detected in sea buckthorn berries.

### 3.3. Ginkgo biloba

*Ginkgo biloba* is one of the most commonly used herbal supplements in the world [79], and is also a crucial source of IGs [80]. It has been demonstrated that *Ginkgo biloba* has various remarkable biological properties, including neuroprotective, anticancer, cardioprotective, and stress-alleviating properties, and could affect tinnitus, geriatric conditions, and psychiatric disorders [81]. The major compounds of *Ginkgo biloba* are terpene lactones and flavone glycosides [82]. Flavonol glycosides are most prevalent in *Ginkgo biloba* leaves, and have been identified as derivatives of the aglycones quercetin, kaempferol, and isorhamnetin, which are, by themselves, present in only small amounts in the leaves. The dominant flavonol glycosides of *Ginkgo biloba* leaves were found to be kaempferol-3-*O*-rutinoside and isorhamnetin-3-*O*-rutinoside (**24**), and content of the latter ranged from 30 to 80 mg/100 g [9].

### 3.4. Pollen Typhae

Pollen Typhae, also known as Pu huang in Chinese, is the dried pollen of *Typha angustifolia*, *Typha orientalis Presl*, or plants of the same genus [83]. Pu huang was acknowledged as a functional food by the National Health Commission of the People’s Republic of China in 2002 [84]. Pollen Typhae has been used as a traditional remedy for analgesia, hemostasis, stranguria, hematuria, and injuries in China. Isorhamnetin-3-*O*-neohesperidoside (**15**) and typhaneoside (**45**), together with other minor flavonoid glycoside congeners, are the main active constituents of pollen Typhae [85]. Isorhamnetin-3-*O*-rhamnosylglucoside (**24**), isorhamnetin-3-*O*-neohesperidoside (**15**) (0.2546~0.3674%), and typhaneoside (**45**) (0.3361~0.5229%) were identified in different pollen Typhae sources [86,87,88].

### 3.5. Calendula officinalis

*Calendula officinalis* is an ornamental, culinary, and valuable herbaceous medicinal plant used medicinally worldwide [89]. It has been widely used as an anti-inflammatory, anticancer, sedative, and antipyretic drug [90]. *Calendula officinalis* is rich in nutrients and contains many terpenes, flavonoids, carotenoids, and lipids [91]. Typhaneoside (**45**) (2.22~5.01 mg/g, DW), narcissin (**24**) (2.10~8.52 mg/g, DW), isorhamnetin-3-*O*-glycoside (**4**) (0.42 ± 0.98 mg/g, DW), and isorhamnetin-3-*O*-(6″-acetyl)-glycoside (**7**) (0.69 ± 3.27 mg/g, DW) were identified in the florets of different varieties of *Calendula officinalis* [42,92]. Isorhamnetin glycosides are considered one of the anti-inflammatory material bases of *Calendula officinalis* [93].

### 3.6. Other Sources

IGs are found in many vegetables, fruits, and medicinal plants. Isorhamnetin-3-*O*-glucoside (**4**) is one of the most abundant flavonoids and is widely distributed in rice varieties [94]. Isorhamnetin-3,7-diglucoside (**18**) is a major flavonoid compound in *Brassica juncea* leaves [95]. IGs have also been detected in *Vaccinium corymbosum* and *Vaccinium myrtillus* [96,97]. Narcissin (**24**) (1.72–5.17 mg/g, DW) was extracted from Microctis folium, which is a commonly used herbal tea material [98,99]. IGs have also been found in different varieties of onion [100,101]. Isorhamnetin-4’-glucoside (**9**) has been reported as a minor flavonoid in onion [23]. *Sambucus nigra*, known as the “elderberry”, has a long history as a medicinal plant [102]. Its extract contains narcissin (**24**) and isorhamnetin-3-*O*-glucoside (**4**), which are capable of regulating glucose and lipid metabolism [103].

## 4. IG Identification and Quantification Methods

Different techniques have been used for the characterization, identification, and quantification of IGs, including spectral techniques and chromatographic techniques. The following review addresses the applicability of the ultraviolet–visible spectrum (UV), infrared spectroscopy (IR), nuclear magnetic resonance (NMR), mass spectrometry (MS), thin-layer chromatography (TLC), high-performance liquid chromatography (HPLC), ultra-performance liquid chromatography (UPLC), and high-speed counter-current chromatography (HSCCC) methods developed for the determination of IGs.

### 4.1. Spectral Techniques and Mass Spectrometry

Various spectral methods have been employed for the identification and quantification of IGs. UV, IR, MS, and NMR have been used to determine the structure of IGs.

#### 4.1.1. UV

The UV absorption spectra of flavonoids mainly have two absorption bands in MeOH, i.e., band Ⅰ, which is caused by the electron transition of the cinnamoyl group, and band Ⅱ, which is caused by the electron transition of the benzoyl group. Regarding UV in flavonols, band Ⅱ absorption usually occurs in the region of 240–280 nm, and is relatively affected by increased hydroxylation of the A-ring; meanwhile, band Ⅰ absorption occurs in the region of 328–385 nm and is relatively affected by increased hydroxylation of the B-ring and C-ring. The addition of diagnostic reagents (NaOMe, NaOAc, NaOAc/H_3_BO_3_, AlCl_3_, and AlCl_3_/HCl) has a certain impact on the UV spectrum [104]. For example, the UV spectrum of isorhamnetin-3-*O*-β-d-galactoside-(1→4)-α-l-rhamnoside-(1→6)-β-d-galactoside (**38**) showed two absorption maxima: 359 nm for band I, and 258 nm for band II. A large bathochromic shift (up to 56 nm) in band I with NaOMe was observed, and was attributed to the presence of free 4′-OH. A free 7-OH group occurred with small bathochromic shift (16 nm) in band II upon the addition of a NaOAc reagent. Additionally, a 5, 7-dihydroxy A-ring was expected to result from the AlCl_3_ and AlCl_3_/HCl UV spectra (λmax nm: 359, 258 (MeOH); 415 (+56), 271 (NaOCH_3_); 403 (+46), 270 (A1Cl_3_); 403 (+46), 268 (AlCl_3_/HCl); 402, 274 (+16) (NaOAc); 364 (+5), 255 (NaOAc/H_3_BO_4_)) [55,105].

#### 4.1.2. IR

IR can be used to determine the characteristic functional groups of IGs. For example, the characteristic functional groups of isorhamnetin-3-*O*-α-l-arabinoside-7-*O*-β-d-glucoside (**26**) isolated from the Callianthemum genus were determined using IR. Its spectrum showed the characteristic absorption bands of a hydroxyl (3444.87 and 3429.43 cm^−1^), a carbonyl (1653.00 cm^−1^), and a phenyl group (1600.92 and 1490.97 cm^−1^) [57]. If the IR spectrum contained a band of 1725 cm^−1^ for ester carbonyl, it indicated that a hydroxyl was acylated [92]. For example, the IR spectrum of isorhamnetin-3-*O*-(6-acetyl-glucoside) (**7**) showed a band at 1725 cm^−1^, which indicated the presence of an ester carbonyl [106].

#### 4.1.3. NMR

NMR is a widely used spectroscopic technique for structure identification. The ^1^H NMR and ^13^C NMR spectra were used to determine chemical shifts in the functional groups and carbon skeleton of IGs.

Strong regularity in the ^1^H NMR spectrum of IGs can be found. The chemistry shifts of H-6 and H-8 of the A-ring are in the ranges 6.00~6.20 and 6.30~6.50 ppm, respectively, and appear as doublets, with a coupling constant of 2.5 Hz, because of two aromatic protons in the meta position. In the B-ring, H-2′, in the range of 7.20~7.90 ppm, appears as a doublet with a coupling constant of 2.5 Hz; H-5′, in the range of 6.70~7.10 ppm, appears as a doublet with a coupling constant of 8.5 Hz; H-6′, in the range of 7.20~7.90 ppm, appears as a doublet of doublets, with coupling constants of 2.5 and 8.5 Hz; and a singlet at 3.80 ppm belongs to 3′-OMe [23,57,107].

Some information on sugar linkage can also be obtained from the ^1^H NMR spectrum. The chemical shift in the H-1 (anomeric) proton varies according to the glycosylation pattern, e.g., 7-*O*-glucosides occurred at 4.8~5.2 ppm, while 7-*O*-rhamnosides occurred at 5.1~5.3 ppm; moreover, 3-*O*-glucosides occurred at 5.7~6.0 ppm, while 3-*O*-rhamnosidesoccurred at 5.0~5.1 ppm [105].

The A ^13^C NMR spectra of IGs can determine the number and environment of each carbon [57]. Moreover, the ^1^H and ^13^C-NMR signals and the linkages of each saccharide can easily be assigned using 2D-NMR, including COSY, HSQC, and HMBC technology. For example, an analysis of the HMQC spectrum of isorhamnetin-3-*O*-α-l-arabinopyranose-7-β-d-glucopyranoside (**26**) can enable all the protons and corresponding carbons in the structure to be assigned. In the HMBC spectrum, correlations between H-1” of arabinose and C-3, and between H-1‴ of glucose and C-7, indicated that arabinose was attached to the C-3 of the aglycone, and glucose was attached to the C-7 of the aglycone, respectively. Thus, they were combined to form isorhamnetin-3-*O*-α-l-arabinopyranose-7-β-d-glucopyranoside (**26**) [107].

#### 4.1.4. MS

MS analysis is based on the mass-to-nucleus ratio and is used to determine molecular structure and weight. The loss of some ion fragments from a molecular or pseudomolecular ion is very characteristic of the mass spectra of IGs.

Electrospray ionization (ESI), an ionization technique, is often used for the MS analysis of IGs. The collision-induced dissociation of a pseudomolecular ion caused a characteristic fragment ion of isorhamnetin glycoside at *m*/*z* 315, which was assigned to isorhamnetin [108]. MS is also used in the determination of the attachment of sugars in IGs. In the mass spectrometry of isorhamnetin-glucoside-di-rhamnoside, a precursor ion at *m*/*z* 769 originated from the product ion at *m*/*z* 315, which is the characteristic ion of isorhamnetin aglycone, and the loss of 454 Da corresponded exactly to two rhamnose units (2 × 146 Da) and one hexose unit (162 Da) [109].

Atmospheric pressure chemical ionization (APCI) is another choice of method for detecting the molecular structure and weight of IGs. The regularities of the characteristic ions of isorhamnetin 3-*O*-glucoside (**4**) obtained in APCI-MS were analyzed; a pseudo molecular ion of *m*/*z* 477 and a second fragment of *m*/*z* 315 were provided, a characteristic fragment ion of *m*/*z* 315 was assigned to isorhamnetin, and the loss of 162 Da corresponded to one glucose unit [108].

Matrix-assisted laser desorption/ionization time-of-flight mass spectrometry (MALDI-TOF MS) is a powerful new technique that can rapidly identify and quantify IGs [110].

### 4.2. Chromatographic Techniques

IGs can be distinguished from each other on the basis of chromatographic techniques. Therefore, the analysis, characterization, and quantification of IGs are usually performed using the following chromatographic techniques: TLC, HPLC, UPLC, and HSCCC.

#### 4.2.1. TLC

TLC is a method that can be used to detect IGs, and has the advantages of rapidity, simplicity, and economy. TLC is usually carried out in ascending mode on standard silica gel plates or microcrystalline cellulose. IGs can be eluted on thin-layer chromatography plates along with the standard compounds and distinguished by their retardation factor (R*_f_*). TLC on silica gel layers for flavonol glycosides is often eluted with an EtOAc-Pyr-H_2_O-MeOH system, an *n*-BuOH–HOAc–H_2_O system, an EtOAc–methyl ethyl ketone–HOAc–H_2_O system, anEtOAc–HOAc–H_2_O system [111], a buthanol–EtOH–H_2_O system [23], or another developing solvent system [107]. Generally, the spots with IGs on a TLC plate can be observed directly under UV light, and the spots are dark. They will appear yellow or green under UV light after the addition of NH_3_ (gas) or a 1:1 mixture of 2% diphenyl-boric acid-ethanolamine complex in EtOH and 10% polethylenglycol 4000 in MeOH stain [112]. Moreover, a 1% ethanolic solution of ferric chloride or aluminum chloride is often used as a TLC dipping solution.

Isorhamnetin-3-*O*-glucoside (**4**) and isorhamnetin-3-*O*-rutinoside (**24**) were detected in the aerial parts of *Peucedanum tauricum* Bieb. TLC separation of the compounds was performed on silica gel plates with two different mobile phases (ethyl acetate–methyl ethyl ketone–formic acid–water, 5:3:1:1, or ethyl acetate–formic acid–water, 9:1:1). The abovementioned compounds were identified by comparing the hR*_f_* (100 × R*_f_*) values with those of standard compounds [111].

#### 4.2.2. HPLC and UPLC

HPLC is suitable for analyzing active components in natural extracts due to its simplicity, sensitivity, precision, and selectivity. In order to identify and quantify IGs, the chromatographic conditions of HPLC mainly include the use of a reverse-phase C_18_ column, acidic water, and MeOH or MeCN as a mobile phase [23,92,113].

HPLC–diode array detection (DAD) coupled with mass spectrometry can be also developed for the analysis of IGs. Narcissin (**24**) (4.9%) and isorhamnetin-3-sophoroside-7-rhamnoside (**43**) (3.7%) were found to be the major flavonoid glycosides in *Hippophae rhamnoides*, and were analyzed ia HPLC-DAD-ESI-MS/MS [114]. The HPLC-DAD-ESI-MS/MS analysis of the *Hippophae rhamnoides* berries of two subspecies provided information on the structure and composition of IGs [10].

Usually coupled with UV, ultraviolet photodiode array, or MS detectors, UPLC is an advanced liquid chromatography technique with the advantages of high resolution, high speed, and high sensitivity [115]. It has become a popular analytical tool for the analysis of many natural compounds, including IGs. Phenolic compounds in sea buckthorn were identified based on UPLC-MS analyses, and it was found that the major compounds contained isorhamnetin-3-*O*-rutinoside (**24**), isorhamnetin-3-*O*-sophoroside-7-*O*-rhamnoside (**43**), isorhamnetin-3-*O*-glucoside (**4**), and isorhamnetin-3-*O*-rhamnoside-glucoside-7-*O*-rhamnoside (**40**) [116]. The berries of *Hippophae rhamnoides* were analyzed via UPLC/PDA/ESI-MS, and it was revealed that their chemical constituents were composed of isorhamnetin-3-neohesperidin (**15**), isorhamnetin-3-glucoside (**4**), isorhamnetin-3-rhamnoside (**3**), isorhamnetin-3-sophoroside-7-rhamnoside (**43**), and free IG in different proportions [77].

#### 4.2.3. HSCCC

High-speed counter-current chromatography (HSCCC), a new, continuous, and efficient liquid–liquid partition chromatography, eliminates the irreversible adsorptive loss of samples onto solid support matrix columns, and has excellent sample recovery compared with certain conventional methods [117,118]. IGs can be separated and purified efficiently through multiple distribution processes using HSCCC. Isorhamnetin-3-*O*-glucoside (**4**) (13 mg) was obtained via one-step HSCCC separation from a 240 mg sample of the medicinal herb lotus plumule [119]. HSCCC was also successfully applied to the preparative isolation of IGs [120].

## 5. The Health-Promoting Effects of IGs

IGs possess a variety of biological properties, including antioxidant, anti-inflammatory, and anti-cancer properties. Research has recently been undertaken to investigate their pharmacological benefits for the treatment of various diseases, such as diabetes, obesity, hepatic diseases, and thrombosis. Their health-promoting effects are summarized below.

### 5.1. Antioxidant Activity

Oxidative damage induced by free radicals results in detrimental outcomes, such as a loss of cellular function and the dysfunction of organic systems [121]. It is worth mentioning that numerous in vitro and in vivo studies have demonstrated the strong antioxidant and radical-scavenging properties of IGs (Table 2).

β-carotene-linoleic acid, 2,2-diphenyl-1-picrylhydrazil (DPPH) scavenging, 2,2′-azino-bis(3-ethylbenzothiazoline-6-sulfonate) (ABTS), oxygen radical absorbance capacity (ORAC), peroxyl radical-scavenging capacity (PSC), superoxide scavenging, peroxynitrite (ONOO(-)) assays, and CUPric reducing antioxidant capacity (CUPRAC) are commonly used indirect assays for identifying antioxidant activity. IGs isolated from the stamens of Nelumbo nucifera showed significant antioxidant activity, as determined via DPPH and ONOO(-) assays [11]. Brassicin (**1**) exhibited stronger free radical-scavenging ability than vitamin C [13] and exhibited DPPH radical- and ONOO(-)-scavenging activity [122]. Isorhamnetin 3-*O*-robinobioside (**22**), isorhamnetin 3-*O*-(2″,6″-*O*-α-dirhamnosyl)-β-galactoside (**37**) [123], typhaneoside (**45**), and isorhamnetin 3-*O*-neohesperidoside (**15**) [124] have been demonstrated to exhibit antioxidant activity using a DPPH radical-scavenging activity assay. Astragaloside (**13**) and narcissin (**24**) possessed antioxidant capacity, which was evaluated using ABST [118]. Narcissin (**24**) and isorhamnetin 3-*O*-rutinoside-7-*O*-glucoside (**47**) exhibited obvious antioxidant activity, which was detected using DPPH, β-carotene-linoleic acid, and ABST [65,125]. Isorhamnetin 3-*O*-neohesperidoside (**15**) was a potent inhibitor of xanthine oxidase and superoxide anion scavengers [126]. Furthermore, researchers have revealed the antioxidant properties of isorhamnetin 3-*O*-glucoside (**4**) and isorhamnetin 3-*O*-galactoside (**8**) in all the antioxidant activity tests employed [127,128,129,130].

Evaluation of the antioxidant properties of IGs were also carried out using various cell type experiments and animal models. The oral administration of isorhamnetin-3,7-diglucoside (**18**) to streptozotocin-induced diabetic rats significantly reduced their levels of 5-(hydroxymethyl) furfural (5-HMF), which is an indicator of the glycosylation of hemoglobin, and of stress [95]. Similarly, isorhamnetin 3-*O*-robinobioside (**22**) exhibited significant antioxidant effects on the human chronic myelogenous leukemia cell line K562 [131]. IGs had the ability to inhibit the formation of H_2_O_2_-induced radicals in the surrounding environment of intestinal epithelial cells [132]. Moreover, the transcriptional genes of the antioxidant system and the DNA repair pathway were upregulated after incubation with isorhamnetin 3-*O*-neohesperidoside (**15**) in pKS plasmid DNA [133]. Narcissin (24) and isorhamnetin 3-*O*-glucoside (**4**) demonstrated strong inhibition of reactive oxygen species (ROS) production in the oxidative burst activity of whole blood, neutrophils, and mononuclear cells [134]. Plant extracts rich in IGs also exhibited antioxidant activity. IG-rich concentrate from *Opuntia ficus-indica* juice had the ability to inhibit the formation of H_2_O_2_-induced radicals in the surrounding environment of intestinal epithelial cells [135]. The total antioxidant activity of *Hippophae rhamnoides* berry extracts, evaluated via ORAC and PSC, was significantly associated with total phenolics, including isorhamnetin-3-rutinoside (**24**) and isorhamnetin-3-glucoside (**4**) [136].

**Table 2:** Antioxidant activity of IGs.

Isorhamnetin Glycosides	Study Model	Method/Assay	Conclusion	Ref.
Isorhamnetin-3-*O*-glucoside (4), Narcissin (24)	/	DPPH, ONOO-	Showed potent antioxidant activity, with IC_50_ values of 11.76 and 9.01 μM in DPPH assay, and 3.34 and 2.56 μM in the ONOO- assay.	[11]
Brassicin (1)	/	DPPH, ABTS	Showed radical-scavenging activity of DPPH radical and peroxynitrite, with IC_50_ values of 13.3 and 2.07 μM.	[13]
Brassicin (1)	/	DPPH, peroxynitrite	Showed radical-scavenging activity of DPPH radical and peroxynitrite, with IC_50_ values of 13.3 and 2.07μM.	[122]
Narcissin (24); isorhamnetin, 3,4′-diglucoside (17)	LPS-induced Raw264.7 mouse macrophage cells	NO	Had an inhibitory effect on the production of NO induced by LPS.	[137]
Isorhamnetin-3-*O*-glucoside (4), 3-*O*-galactoside (8)	β-carotene- linoleic acid	DPPH, ABTS, CUPRAC	Act as free radical scavengers and chain-breaking antioxidants of DPPH, with IC_50_ values of 4.84 and 4.51 μM.	[127]
Isorhamnetin 3-*O*-galactoside (8)		DPPH	Showed high antioxidant activity compared to Trolox (standard antioxidant compound).	[128]
Typhaneoside (45); isorhamnetin-3-*O*-neohesperidoside (15)	HUVECs treated with LPS	NO, MDA, SOD	Reduced levels of MDA, increased SOD activity and NO bioactivity.	[124]
Isorhamnetin 3-*O*-robinobioside (22)	K562 cell line induced by H_2_O_2_	CAA	Inhibited oxidation (IC_50_ = 0.225 mg/mL) and genotoxicity (by 80.55% at 1000 μg/mL).	[131]
Isorhamnetin 3-*O*-robinoside (22); isorhamnetin 3-*O*-(2″,6″-*O*-α- dirhamnosyl)-β-galactoside (37)	/	DPPH	Effectively scavenged DPPH radicals, with IC_50_ values of 3.8 and 4.3 μM.	[123]
Isorhamnetin-3-*O*-glucoside (4)	/	DPPH, ABTS, FRAP	Highly correlated with DPPH, ABTS, and FRAP (r = 0.672, r = 0.660, r = 0.943, respectively).	[130]
Astragaloside (13), narcissin (24)	/	ABTS	Possessed antioxidant capacity, with IC_50_ values of 33.43 and 40.97 μg/mL.	[118]
Narcissin (24); isorhamnetin 3-*O*-glucoside (4)	/	DPPH	Showed pronounced antioxidant activity, with IC_50_ values of 165.62 and 177.91 μg/mL.	[65]
Narcissin (24); isorhamnetin-3-*O*-rutinoside-7-*O*-glucoside (47)	/	DPPH, ABTS	Showed obvious antioxidant activity.	[125]
Narcissin (24)	HepG2 cells	CAA	Showed significant in vitro antioxidant activity, with CAA value significantly correlated with narcissin (24) (R^2^ = 0.998).	[136]
IGs	H_2_O_2_-induced intestinal epithelial cells	ORAC	Able to counteract protein oxidation.	[132]
Isorhamnetin 3-*O*-neohesperidoside (15)	Hydroxyl radical-induced DNA damage pKS plasmid	MDA, DNA-strand scission assay	Transcriptions of several genes related to the antioxidant system (HMOX2 and TXNL) were upregulated.	[133]
Isorhamnetin 3-*O*-neohesperidoside (15)	/	ABTS, xanthine/xanthine oxidase	Was a potent inhibitor of xanthine oxidase (IC_50_ = 48.75 μg/mL) and superoxide anion scavengers (IC_50_ = 30 μg/mL).	[126]
Isorhamnetin 3-*O*-galactoside (8)	/	ABTS	Showed ABTS radical-scavenging activity (IC_50_ = 6 ± 0 μM).	[129]
Narcissin (24); isorhamnetin 3-*O*-glucoside (4)	Whole blood, neutrophils, or monocytes	ROS	Demonstrated potent inhibition of ROS production.	[134]

### 5.2. Anti-Inflammatory Activity

IGs have anti-inflammatory properties due to different mechanisms. As an important inflammatory mediator, high-mobility-group protein 1 (HMGB1) contributes to organ damage and inflammation [138]. Isorhamnetin 3-*O*-galactoside (**8**) (5 μM) has been demonstrated to significantly inhibit the release of HMGB1 and reduce HMGB1-dependent inflammatory responses in human endothelial cells. It was found that **8** (4.8 mg/mouse) could also inhibit HMGB1 receptor expression, the HMGB1-mediated activation of NF-kB, and the production of tumor necrosis factor (TNF-α) in mice [139].

Mitogen-activated protein kinase (MAPK) signaling pathways, including p38, c-Jun *N*-terminal kinase (JNK), and extracellular regulated kinases (ERK), play crucial roles in inflammatory responses [140]. Isorhamnetin 3-*O*-galactoside (**8**) (50 μM) reduced cecal ligation and endothelin C receptor perforation-mediated shedding and down-regulated the phosphorylation of p38 MAPK, ERK 1/2, and JNK [14]. Similarly, isorhamnetin 3-*O*-glucuronide (**5**) exhibited anti-inflammatory activity by increasing heme oxygenase-1 (HO-1) expression and suppressing the JNK and p38 signaling pathways in LPS-induced RAW264.7 macrophage cells [141]. Moreover, isorhamnetin 3-*O*-glucuronide (**5**) inhibited the production of ROS (10 μM), as well as the release of elastase, in a human neutrophil model (1 μM) and suppressed the upregulation of inducible nitric oxide synthase (iNOS) expression (5 μM), and could be considered to display anti-inflammatory activity [46,142].

Many studies have shown the anti-inflammatory properties of IGs by inhibiting inflammatory cytokines. The inflammatory activity of narcissin (**24**) (100 μM) and isorhamnetin 3-*O*-glucoside (**4**) (100 μM) was mediated via the inhibition of nuclear factor kappa-B (NFκB) and inflammatory mediators such as TNF-α, interleukin-1β (IL-1β), and interleukin-6 (IL-6) in phytohaemagglutinin-stimulated human peripheral blood mononuclear cells (PBMC) [132]. Likewise, narcissin (**24**) (40 μM) achieved the inhibition of inflammatory cytokines (TNF-α, IL-1β, and IL-6) in advanced glycation end product (AGE)-induced RAW264.7 cells [143]. Isorhamnetin-3-*O*-[2,3-*O*-isopropylidene-α-l-rhamnopyranosyl]-(1→6)-*O*-β-d-glucopyranoside (**11**) (25 μM) showed a significant inhibitory effect on NO release and the secretion of the cytokines IL-6 and TNF-α [48]. Isorhamnetin-3,4′-diglucoside (**17**) (100 μg/mL) and isorhamnetin 3-*O*-glucoside (**4**) (100 μg/mL) have shown the inhibitory effect of IL-6 production on TNF-α-stimulated human osteosarcoma MG-63 cells [144]. Isorhamnetin 3-O-glucoside (**4**) (100 μg/mL) showed distinct anti-inflammatory activity with no toxicity on RAW 264.7 macrophage cells as compared to dexamethasone [145]. Seddik Ameur et al. studied the anti-inflammatory activity of IGs extracted from *Opuntia ficus-indica* flowers, and their results showed that isorhamnetin-3-*O*-robinobioside (**22**) is the product responsible for the anti-inflammatory activity [146]. Both Opuntia ficus-indica extract (OFI-E) and isorhamnetin-3-*O*-rhamnosylglucoside (**24**) (125 ng/mL) significantly inhibited cyclooxygenase-2 (COX-2), TNF-α, and IL-6 production, of which 24 compounds have been suggested to be suitable natural compounds for the development of a new anti-inflammatory ingredient [147]. The total flavonoid-rich IGs from sea buckthorn exhibited a protective effect against LPS/CS-induced airway inflammation by inhibiting the ERK, PI3K/Akt, and PKCα pathways and diminishing the expression of IL-1β, IL-6, and COX2 in mice [148].

### 5.3. Anti-Cancer Activity

Flavonoids have great potential for anticancer prevention [149]. IGs have also been proven to possess anticancer effects. Brassicin (**1**) (22.8 µg/mL) showed in vitro cytotoxicity against human colon cancer cells in the HCT116 cell line [150]. Isorhamnetin 3-*O*-neohesperidoside (**15**) (2.47 μg/mL) showed potent cytotoxicity against breast ductal carcinoma and colorectal adenocarcinoma (Caco-2) cells [151]. Narcissin (**24**) showed cytotoxic effects in Hela cells and the hormone dependent prostate carcinoma LNCaP cell line (IC_50_ = 20.5 μg/mL) [152,153].

**Figure 4:**
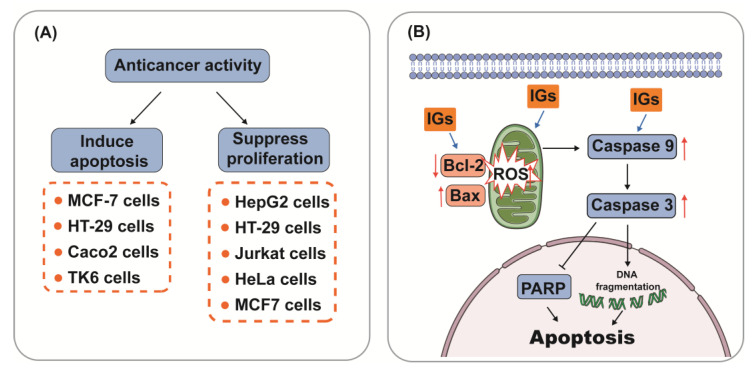
Anticancer activity (**A**) and mechanism of regulating the apoptotic pathway (**B**) of IGs.

Mechanically, IGs have been involved in the induction of apoptosis and the inhibition of cancer cell proliferation (Figure 4A). Apoptosis, the most vital cell death mechanism, ultimately contributes to tumor progression [154]. Mitochondria play an essential role in cell death signaling and ROS generation [155]. The production of ROS above a threshold level can trigger apoptosis in cancer cells, thereby limiting further cancer progression [156]. After the excessive production of ROS, the expression of genes related to the mitochondrial apoptosis pathway (Bax, Caspase9, and Caspase3) was aggravated, and the expression of the anti-apoptotic gene Bcl-2 was reduced [157]. Emerging evidence suggests that IGs promote ROS generation and the activation of mitochondria-dependent apoptosis in cancer cells (Figure 4B). Isorhamnetin-3-*O*-β-d-glucuronide (**5**) (25–100 μΜ) dose-dependently exhibited a strong cytotoxic effect through the ROS-dependent apoptosis pathway in the human breast cancer cell line MCF-7 [158]. In xenografted immunosuppressed mice, *Opuntia ficus-indica* extract (OFI-E) and isorhamnetin-3-*O*-glucosyl-rhamnoside (**28**) reduced tumor growth through the overexpression of cleaved Caspase-9, Hdac11, and Bai1 proteins. Moreover, OFI-E reduced the expression of Bcl-2 [159]. IGs from opuntia ficus-indica pads were cytotoxic against HT-29 cells (IC_50_ = 4.9 ± 0.5 μg/mL) and Caco-2 cells (IC_50_ = 8.2 ± 0.3 μg/mL) as they induced apoptosis [160]. Isorhamnetin-3-*O*-rhamnosylglucoside (**24**) induced cell death in the human colon cancer cell line HT-29 (10 μg/mL) through an increase in the Bax/Bcl-2 ratio, indicating that **24** induced apoptosis through mitochondrial damage [15]. Isorhamnetin 3-*O*-robinobioside (**22**) enhanced the apoptosis effects in tested human lymphoblastoid TK6 cells, which were confirmed via DNA fragmentation and PARP cleavage, indicating the release of caspase-3 [161]. Numerous studies show the beneficial effects of IGs and their capability for suppressing proliferation in cancer cells. Ana et al. extracted natural extracts from *Opuntia ficus-indica* and *Opuntia robusta* (ED_50_ value < 0.5 mg GAE/mL) residues, and evaluated their anti-proliferative effects in human colon cancer HT29 cells. Their results verified that IGs inhibited cell growth and induced cell cycle arrest at different checkpoints (G1, G2/M, and S) [162]. Isorhamnetin-3-*O*-rhamnosylglucoside (**24**) (394.68 ± 25.12 μM) inhibited the proliferation of chronic myelogenous leukemia cells [163,164]. Isorhamnetin 3-*O*-2′′′′-*O*-acetyl−β-d-xylopyranosyl-(1→6)-[β-d-apiofuranosyl-(1→2)]-β-d-glucopyranoside (**36**) (IC_50_ = 57/42/59 μM) and isorhamnetin 3-*O*-2‴-*O*-acetyl−β-d-xylopyranosyl-(1→6)-β-d-glucopyranoside (**10**) (IC_50_ = 71/60/67 μM) were investigated for their potential cytotoxic activity in three cancer cell lines (Jurkat cells, cervical carcinoma cells, and MCF7 cells) and showed moderate antiproliferative activity [27].

Furthermore, isorhamnetin 3-*O*-glucoside (**4**) (10 μM) exerted its inhibitory effects on matrix metalloproteinase-9 and -2 in HT1080 human fibrosarcoma cells by interfering with activator protein-1 transcription factor binding [165]. Isorhamnetin-3,7-diglucoside (**18**) (50–100 μg/mL) induced a 20% decrease in cancer intestinal cell survival through glycogen synthase kinase 3-beta regulation in intestinal cells [166].

### 5.4. Hepatoprotective Ability

The liver is the most essential and functional organ in the body, and it is where primary detox and metabolic events occur [167]. Liver injury can be caused by various factors, including alcohol, microbial infection, drugs, biological toxins, and chemical agents [168]. Flavonoids in many different foods and medicinal plants have therapeutic potential in liver disease [169].

**Figure 5:**
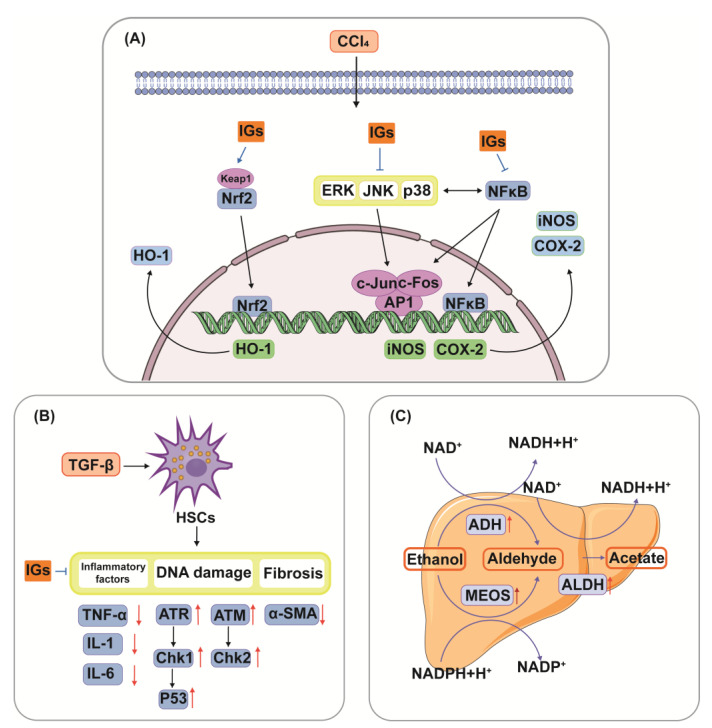
Hepatoprotective mechanism of IGs. Networks of molecular signaling underlying anti-oxidative stress and anti-inflammatory effects of IGs in CCl_4_-induced hepatic damage(**A**). IGs inhibit TGF-β-induced activation of HSCs through the DNA damage pathway (**B**). Hepatic metabolic pathways through which IGs alleviate the adverse effects of ethanol (**C**).

Studies have confirmed that IGs play an important role in liver injury by modulating multiple pathways (Figure 5). The hepatoprotective effects of IGs are closely linked with their antioxidant and anti-inflammatory effects. Isorhamnetin 3-*O*-galactoside (**8**) (100 mg/kg) reduced serum TNF-α levels, aminotransferase activities, and the hepatic level of malondialdehyde (MDA); attenuated increases in iNOS and COX-2 protein and mRNA expression levels; attenuated increases in nuclear factor kappa-B (NF-κB) and c-Jun nuclear translocation; and augmented the levels of HO-1 and mRNA expression and the nuclear level of nuclear factor E2-related factor 2 (Nrf2) in a carbon tetrachloride (CCl_4_)-induced hepatic damage model (Figure 5A). This suggests that IGs exhibit hepatoprotective effects by enhancing the antioxidative defense system and reducing the inflammatory signaling pathways [16]. A similar result was obtained for the hepatoprotective effects of isorhamnetin 3-*O*-glucoside (**4**) (20 μg/mL/mouse). It suppressed the increase in plasma alanine aminotransferase (ALT) and aspartate aminotransferase (AST) activities in CCl_4_-induced liver injury mice [170]. *Opuntia ficus-indica* fruit juice (3 mL/rat) administration exerted protective and curative effects against the CCl_4_-induced degenerative process in rat liver [171]. The oral administration of a phenolic-rich fraction of sea buckthorn leaves (25–75 mg/kg) significantly protected against CCl_4_-induced elevation in AST, ALT, c-glutamyl transpeptidase, and bilirubin in the serum, and also protected against histopathological changes produced by CCl_4_, such as hepatocytic necrosis, fatty changes, and vacuolation [172]. In another study, typhaneoside (**45**) exhibited hepatoprotective effects on D-GalN-induced cytotoxicity in primary cultured mouse hepatocytes [173]. The phytochemical constituents of cactus branch extract (92 mg/kg), which were found to possess excellent antioxidant properties, had protective effects against lithium-induced hepatotoxicity and oxidative stress in rats [174].

IGs also had an improvement effect on hepatic lipid accumulation. In high-fat diet-fed mice, OFI-E (0.3%, 0.6%) reduced fatty acid synthesis and increased fatty acid oxidation and caused a decrease in hepatic fat accumulation, thereby preventing hepatic steatosis [70]. Isorhamnetin-3-*O*-glucoside (**4**), isorhamnetin, 3,4′-diglucoside (**17**), and isorhamnetin 3-*O*-β-d-glucopyranosyl-7-*O*-β-d-gentiobioside (**47**) (30 µM) had significant inhibitory effects on sodium oleate-induced triglyceride overloading in HepG2 cells [53]. Furthermore, biochemical and histopathological studies showed that sea buckthorn flavonoids (200 mg/kg, po) significantly improved biomarkers in the serum and liver of tetracycline-induced nonalcoholic fatty liver mice [175].

Zhang G et al. observed that isorhamnetin-3-*O*-β-d-glucopyranoside-7-*O*-α-l-rhamnoside (**20**) (40 μM) exhibited a profound inhibitory effect on the activation of hepatic stellate cells (HSCs) induced by transforming growth factor-β (TGF-β), and decreased the levels of inflammatory factors. It over-regulated the proteins of the DNA damage signaling pathway, including the ataxia telangiectasia mutated gene (ATM), Rad3-related gene (ATR), checkpoint kinase1 (Chk1), checkpoint kinase2 (Chk2), p53, and alpha-smooth muscle actin (α-SMA) (Figure 5B) [176]. In addition, the active components of sea buckthorn berry (20 and 40 mg/kg) had inhibitory effects on the development of fibrosis in rats after bile duct ligation, and they attenuated liver injury and inflammation by downregulating the expression of αSMA, while over-regulating the DNA damage signaling pathways and their related genes.

Isorhamnetin 3-*O*-β-d-glucopyranoside (**4**) alleviated the adverse effect of ethanol ingestion by enhancing the activities of alcohol dehydrogenase (ADH), the microsomal ethanol oxidizing system (MEOS), and aldehyde dehydrogenase (ALDH) in a hepatic alcohol-metabolizing enzyme system in rats (Figure 5C) [177]. In addition, sea buckthorn fermentation liquid (1.75, 2.675, 5.35 g/kg) protected against alcoholic liver disease and modulated the composition of the gut microbiota. It lowered ALT, AST, TNF-α, MDA, and IL-6, while modulating the gut microbiota composition [178].

### 5.5. Antidiabetic Activity

The antidiabetic properties of IGs may appear through different functions. IGs inhibit various pathways associated with the progression of diabetes, including the regulation of glucose metabolism and enhancing insulin secretion [179].

**Figure 6:**
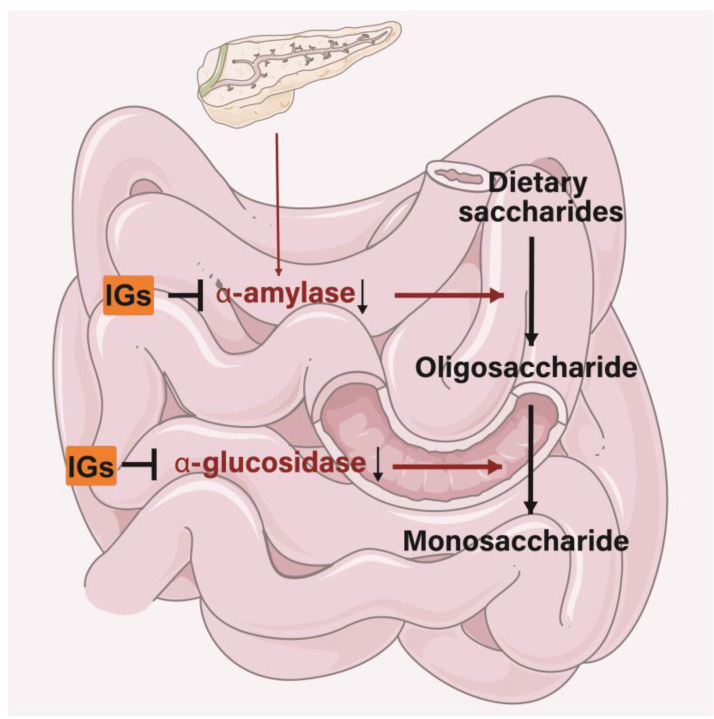
Mechanism of IGs inhibiting α-amylase and α-glucosidase.

IGs exert inhibitory activity on several enzymes involved in diabetes management. In the small intestine, IGs inhibit the activity of α-amylase and α-glucosidase, thereby reducing the conversion of dietary saccharides into easily absorbed monosaccharide, and thus, reducing the postprandial enhancement of blood glucose levels (Figure 6). Isorhamnetin-3-*O*-glucoside (**4**) showed a strong ability to bind to α-amylase and α-glucosidase (the IC_50_ values were 0.16 ± 0.06 and 0.09 ± 0.01 µM) [180]. Narcissin (**24**) (IC_50_ = 0.129 mM) could be useful in lowering postprandial blood glucose by inhibiting α-amylase activity [181]. Meanwhile, 24 was a good 15-lipoxygenase (IC_50_ = 45 ± 2 µM) inhibitor [182,183]. Isorhamnetin glucosyl-rhamnosyl-pentoside (50 μg/mL) was reported to exhibit antihyperglycemic activity by inhibiting α-amylase activity [184]. Sea buckthorn aqueous extracts were correlated with lipase/α-amylase inhibitory activity in all phases of a digestion model in vitro, with gastric and intestinal fractions largely inhibiting enzyme activity [185].

Dipeptidyl peptidase-IV (DPP-IV) inhibitors promote insulin secretion by prolonging the activities of incretin glucagon-like peptide 1 and glucose-dependent insulinotropic polypeptide [186]. In vitro experiments showed that isorhamnetin 3-*O*-glucoside (**4**) (IC_50_, 6.53 ± 0.280 μM) and isorhamnetin 3-*O*-rutinoside (**24**) (IC_50_, 8.57 ± 0.422 μM) had strong inhibitory effects on DPP-IV, which may provide new insights into isorhamnetin glucosides as DPP-IV inhibitors for controlling blood glucose [187]. The inhibition of protein tyrosine phosphatase 1B (PTP1B) activity increased insulin sensitivity and reduced blood glucose levels [17]. In vitro, **4** (IC_50_, 1.16 ± 0.03 μM) and **24** (IC_50_, 1.20 ± 0.05 μM) exhibited potent inhibitory activity against PTP1B, revealing that they could be potential anti-diabetic drugs [188].

Moreover, IGs improved the secondary complications of diabetes. In diabetes, the overexpression of aldose reductase induces the conversion of glucose to sorbitol via the polyol pathway, thereby inducing complications of diabetes, such as neuropathy, nephropathy, and retinopathy [189]. Isorhamnetin-7-*O*-β-neohesperidoside (**12**) (IC_50_ = 5.45 ± 0.26 µg/mL) and isorhamnetin 3-*O*-glucoside (**4**) (IC_50_ = 21.55 ± 1.52 µg/mL) exhibited remarkable aldose reductase inhibition activity [12]. It was also found that **4** (25 mg/kg) inhibited rat lens aldose reductase and sorbitol accumulation in streptozotocin-induced diabetic rat tissues [190]. Isorhamnetin 3-*O*-α-l-rhamnopyranosyl-(1→6)-β-d-glucopyranoside (**24**) (IC_50_ = 9 μM) was determined to exhibit a high degree of rat lens aldose reductase inhibitory activity in vitro [191].

### 5.6. Anti-Obesity Activity

Flavonoids could protect against obesity-related pathology by inhibiting adipogenesis and exerting anti-inflammatory activity [192]. Sea buckthorn leaf extract contains a high content of flavonoid glycosides, especially isorhamnine-3-glucoside (**4**) and quercetin-3-glucoside [78]. Flavonoid glycosides extracted from sea buckthorn leaves (SLGs) could suppress diet-induced obesity in C57BL/6J mice [98]. In this study, the authors mentioned that 12 weeks of oral administration with a high-fat diet (HFD, 60 kcal% fat) + 0.04% (*w*/*w*) SLGs significantly prevented adiposity and dyslipidemia by suppressing lipogenesis and the absorption of dietary fat. This anti-obesity effect was explained by the improvement of inflammation and a decrease in gluconeogenesis. Narcissin (**24**) and **4** (30 μM) showed moderate inhibitory effects on triglyceride and glycerol-3-phosphate dehydrogenase activity in a 3T3-L1 preadipocyte [193]. Furthermore, it was demonstrated by Chang-Suk Kong et al. that **4** (20 μM) potently suppressed adipogenic differentiation by downregulating peroxisome proliferator-activated receptor-γ, CCAAT/enhancer-binding proteins, sterol regulatory element-binding protein 1, and the adipocyte-specific proteins in 3T3-L1 preadipocytes. Furthermore, the specific mechanism mediating its action occurred through the activation of AMPK [194].

IG-rich plant extracts also have obvious anti-obesity effects. César Rodríguez-Rodríguez et al. have demonstrated that oral treatments of HFD, with a low (0.3%) or high (0.6%) dose of OFI-E rich in isorhamnetin glycosides, to C57BL/6 mice for 12 w ameliorated the development of HFD-induced obesity-related metabolic abnormalities by reducing weight gain, increasing insulin secretion, and enhancing energy expenditure in mice [70]. Further mechanistic studies verified that OFI-E and IGs could reduce fatty acid synthesis and increase fatty acid oxidation, leading to reduced fat accumulation in adipose tissue, thereby preventing adipocyte hypertrophy. OFI-cladode infusions (1%, administered daily in the drinking water) reduced proinflammatory cytokines such as TNF-α, IL-1β, and IL-6 in the colon, adipose tissue, and spleen in Swiss male mice fed an HFD, as well as IL-6 and TNF-α in the plasma. These results suggested that OFI-cladode ameliorated HFD-induced obesity-related inflammation [195]. The results showed that intragastric administration of the extract from *Hippophae rhamnoides* seeds with concentrations of 100 and 300 mg/kg led to anti-obesity, triglyceride-lowering, and hypoglycemic effects in obese mice. It markedly inhibited macrophage infiltration into adipose tissue by regulating PPARγ and PPARα gene expression and inhibiting adipose tissue inflammation [196]. Oral sea buckthorn flavonoid administration (0.06% and 0.31% *w*/*w*, mixed in the diet) was able to alleviate body weight gain and insulin resistance in high-fat- and high-fructose-diet-induced C57BL/6J mice [197]. An extract of black soybean leaves (EBL), which mainly contains quercetin glycosides and isorhamnetin glycosides, inhibited HFD-induced obesity. Dietary supplements with 1% (wt/wt diet) EBL significantly reduced weight gain, improved glucose homeostasis, and decreased the glucose, insulin, HbA1c, and HOMA-IR index levels in HFD-fed mice. Mechanistic studies revealed that EBL inhibited hyperglycemia and hepatic steatosis through the adiponectin and AMPK signaling pathways, while isorhamnetin 3-*O*-α-l-rhamnopyranosyl (1→2)]-β-d-galactopyranosid (**33**) (50 μM) directly reduced lipid accumulation in HepG2 cells by enhancing AMPK activity [62].

### 5.7. Antithrombotic Activity

Thrombosis is a critical event in diseases correlated with atherosclerosis, myocardial infarction, and stroke [198]. The aggregation of platelets at the site of injury, as well as thrombin generation and fibrin formation triggered by the activation of tissue factors, are involved in thrombosis formation [199]. Therefore, the therapeutic mechanism includes the inhibition of platelet activation, adhesion, and aggregation, the improvement of fibrinolytic system function, and the regulation of coagulation system function [200].

Sae-Kwang Ku et al. assessed the antithrombotic activity of isorhamnetin 3-*O*-galactoside (**8**) from *Oenanthe javanica*. Studies have confirmed that it (10 μM) could significantly prolong the activated partial thromboplastin time and prothrombin time, inhibit the activity of thrombin and factor X, and inhibit the thrombin in human umbilical vein endothelial cells activated by TNF-α and the generation of factor X. In addition, isorhamnetin 3-*O*-galactoside (2.5 mg/kg) also elicited consistent anticoagulant effects in mice [201]. IGs isolated from sea buckthorn fruits showed marked anticoagulant and antiplatelet activity [202]. A thrombus-formation analysis system indicated that isorhamnetin 3-*O*-β-glucoside-7-*O*-α-rhamnoside (**20**) (50 µg/mL) and isorhamnetin 3-*O*-β-glucoside-7-*O*-α-(3‴-isovaleryl)-rhamnoside (**34**) (50 µg/mL) demonstrated anti-coagulant potential in whole blood. BartoszSkalski et al. came to the consistent conclusion that isorhamnetin 3-*O*-β-glucoside-7-*O*-α-(3‴-isovaleryl)-rhamnoside (**34**) (5, 10 µg/mL) possessed anti-platelet and anticoagulant properties, which extended the thrombin time and inhibited aggregation induced by thrombin [69]. Isorhamnetin 3-*O*-rhamnosylglucoside (**24**) (0.4 mg/mL) can stimulate the endothelial cell to produce tissue plasminogen activators and prostaglandins and possesses antithrombotic properties [87]. Isorhamnetin-3-*O*-α-l-rhamnoside-(1→2)-β-d-glucoside (**15**) isolated from pollen Typhae can also stimulate porcine aortic endothelial cells to produce tPA, and it was revealed that it has antithrombotic effects. Sae-Kwang Ku et al. demonstrated that isorhamnetin-3-*O*-galactoside (**8**) (10 μM) inhibited the TNF-α-induced production of plasminogen activator inhibitor type 1 (PAI-1) and reduced the ratio of PAI-1 to tissue-type plasminogen activator (tPA) [201].

### 5.8. Toxic Effects

Flavonoids are natural components of fruits, vegetables, tea, wine, traditional medicines (such as *ginkgo biloba*), and a considerable number of herbal dietary supplements. With growing interest in alternative medicine, the general population is consuming more flavonoids [203]. Since flavonoids are common edible ingredients in our daily diets, research on their potential cytotoxicity is warranted.

Currently, there are no systematic toxicological studies on IGs, and further studies are needed. Bee bread (BB) is a fermented mixture of plant pollen, honey, and bee saliva, and is rich in flavonoid glycoside derivatives [204]. Filipa Sobral et al. collected a variety of BB samples, and the most abundant compounds in BB1 (>400 µg/mL) were isrohamnetin-*O*-hexosyl-*O*-rutinoside and isorhamnetin-*O*-pentosyl-hexoside. They found that the BB1 sample showed no toxicity to non-tumor porcine liver primary cells [205]. Isorhamnetin-3-rutinoside-4′-glucoside (**35**), isolated from *P. lanceolata* inflorescences, showed significantly less cytotoxicity towards the nontumorigenic cell line MCF-12A at a concentration of 400 µM [206]. Isorhamnetin-3-*O*-β-d-galactopyranoside (**8**) and isorhamnetin-3-*O*-β-d-glucopyranoside (**4**) (100 µg/mL) isolated from *Salsola imbricata Forssk.* exhibited no cytotoxicity in RAW 264.7 macrophage cells [158]. Furthermore, it was demonstrated that the viability of PBMCs was slightly decreased after 48 h of incubation with isoretin-3-*O*-rutin (**24**) (0–180 µM) from *Cyrtosperma johnstonii*. However, the decrease in cell viability was no greater than 30% [207]. A brine shrimp toxicity assay of extracts and isolated compounds from *Terminalia macroptera* leaves showed that narcissin (**24**) was not toxic against brine shrimp larvae at the tested concentrations (200 µM) [182].

## 6. Bioaccessibility of IGs

The bioaccessibility of bioactive compounds refers to the maximum fraction of the compound released from the food matrix into the lumen of the gastrointestinal tract to be absorbed [208]. Most flavonoids exist in nature as glycosides, in which sugar residues modify the absorption mechanism and their ability to enter cells or interact with transporters and cellular lipoproteins [209,210]. Flavonoid glycosides exhibit better bioavailability both in vitro and in vivo, which is probably due to their higher aqueous solubility and stability during digestion [8]. At the same time, the gut microbiota plays an important role in improving the bioavailability and enhancing the absorption of flavonoids [211]. The deglycosylation of flavonoid glycosides by the gut microbiota enhances the bioavailability of flavonoids [212].

Compared with isorhamnetin aglycone, IGs have higher accessibility. Antunes-Ricardo et al. found that glycosylation protected isorhamnetin from degradation during simulated digestion, and IGs were better retained in the circulatory system than aglycone [8]. Isorhamnetin-3-*O*-rutinoside (**24**) (93.2 ± 0.2%) and isorhamnetin 3-*O*-glucoside (**4**) (66.8 ± 1.7%) from almond skins showed higher bioaccessibility than isorhamnetin (25.1 ± 7.0%) after simulated digestion [213]. Isorhamnetin glucosyl-rhamnosyl-rhamnoside, isorhamnetin glucosyl-rhamnosyl-pentoside, isorhamnetin hexosyl-hexosyl-pentoside, and isorhamnetin glucosyl-pentoside showed high bioaccessibility in the peels of four prickly pear varieties during in vitro simulated gastrointestinal digestion [214]. Isorhamnetin glucosyl-rhamnosyl-rhamnoside and isorhamnetin glucosyl-pentoside in *Opuntia ficus-indica* cladodes showed bioaccessibility values of 58% and 38% [215].

It was also reported that the antidiabetic, anti-inflammatory, and antiallergic activities of flavonoid glycosides were similar or even higher than those of aglycones when provided orally [216,217,218,219]. The effect of flavonoid glycosides is beneficial, probably due to the fact that flavonoid glycosides maintain higher plasma concentrations and have a longer mean residence time in the blood than aglycones [220]. Typhaneoside (**45**) and isorhamnetin-3-*O*-neohesperidoside (**15**) were detected immediately after the oral administrations of pollen typhae extract in rats, indicating that they were rapidly absorbed after oral administration [86,221]. IGs in sea buckthorn berries were monoglucuronidated in humans and were readily bioavailable [222]. Following the ingestion of lightly fried onions, flavonols were absorbed into the plasma of humans as glycosides, with a higher accumulation of isorhamnetin-4′-glucoside (**9**) in the plasma and urine than quercetin conjugates, which indicated that 9 may be preferentially absorbed [223]. Similarly, the results of a randomized crossover supplementation trial in female volunteers showed that 9 underwent significant elevation in the plasma after the ingestion of onion powder [224]. Antunes-Ricardo et al. reported that IGs found naturally in O. ficus-indica have a longer elimination half-life than isorhamnetin, suggesting that they can maintain constant plasma concentrations, and thus, prolong their biological effects [8].

Planar lipophilic polyphenols, such as curcumin, epigallocatechin gallate, quercetin, and genistein, are known as Pan-Assay Interference Compounds (PAINS) or Invalid Metabolic Panaceas (IMPS) because of their ability to interfere with membrane dipole potential [225]. Ana Marta de Matos et al. demonstrated that compounds produced via *C*-glycosylation are no longer able to alter the membrane dipole potential [226]. However, *O*-glycosylated compounds are easily hydrolyzed in the gut, so they are not suitable for this strategy. There are no more studies on the interference of isorhamnetin glycosides on membrane dipole potential, so further research in this field is warranted.

## 7. Marketed Products Related to IGs

In recent years, there has been increased interest in natural phytonutrients. Phytonutrients, such as beta-carotene (representative food, e.g., carrots), lutein (collard), isoflavones (soybeans), resveratrol (red wine), and anthocyanins (grapeseed), are known to provide a variety of significant benefits to humans and improve human well-being [227]. IGs as phytonutrients have been used in food and as a remedy against different health disorders, and processed into various products.

### 7.1. Food and Functional Food Products Using Opuntia ficus-indica

The cultivation for *Opuntia ficus-indica* is scattered across various parts of the world, such as Central and South America, Southern Spain, the Mediterranean Sea, Angola, Australia, India, and South Africa [228,229,230]. *Opuntia ficus-indica* has long been marketed in different forms, such as fresh, frozen, or pre-cooked, and used as fresh greens and in salads in Mexico, Latin America, South Africa, and the Mediterranean area [231]. As a popular dietary supplement in the United States, *Opuntia ficus-indica* products could be potentially utilized for body weight control and liver function support.

*Opuntia ficus-indica* can be processed into many food products (Figure 6). Its cladodes have been used as a vegetable, usually eaten freshly peeled, in salads, cooked (boiled, fried, or deep-fried), or made into a juice or sauce [232,233]. Its fruit can be squeezed and used to produce juices, jams, candies, beverages, ice creams, and teas [234,235,236], and has also been added to rice field bean flour to produce an innovative gluten-free pasta [237]. Its peel has been utilized as a substitute for vitamin E, as an antioxidant in margarine preservation [238]. Its seed can be used to make oil [239]. Freeze-dried pulp can be added to rice or corn flour, resulting in a puffed flavanol-rich snack [240]. Its cladodes, pulp, or seeds, or whole plant, can be made into flour, which can partly substitute wheat or corn flour in doughs, bread, cookies, snacks, or desserts [18,241,242]. *Opuntia ficus-indica*-related products on the market have been listed in Table 3.

During the processing of *Opuntia ficus-indica* products, the processing technology used preserves the fruit’s nutritional and sensory characteristics, and increases the content of IGs. It was reported that the extrusion or the preparation of concentrated juice pretreated with a pulsed electric field of *Opuntia ficus-indica* allowed for an increase in isorhamnetin glycoside content, especially isorhamnetin-3-*O*-rutinoside (**24**) [243,244].

### 7.2. Food and Functional Food Products of Hippophae rhamnoides

*Hippophae rhamnoides* possesses abundant bioactive compounds that can be utilized in the preparation of functional food products [19]. The berries, seeds, leaves, and even bark can be processed into supplemental products that gave the body all-natural assistance for many different functions. *Hippophae rhamnoides* leaves have gradually begun to be used in the food industry for tea processing [245]. A wide variety of products—jams, jellies, juices, powder, and seed oils—can be formulated from *Hippophae rhamnoides* berries [76]. Over the years, *Hippophae rhamnoides* products have increased in popularity (Table 4) [246]. *Hippophae rhamnoides* product consumption as part of the regular diet is common in Asia, the United States, and some European countries [247].

It was found that isorhamnetin derivatives were the most important flavonoids in *Hippophae rhamnoides* fruit juice [248]. The treatment of by-products in juice production via solvent-free microwave hydrogenation diffusion and gravity technology obtained more flavonoids, such as isorhamnetin, isorhamnetin 3-*O*-glucoside (**4**), isorhamnetin 3-*O*-rutinoside (**24**), than conventional solvent extraction [249].

## 8. Conclusions and Prospects

IGs are bioactive flavonoids found in various plants, such as *Opuntia ficus-indica*, *Hippophae rhamnoides*, and *Ginkgo biloba*. Routine and innovative assay methods, such as IR, TLC, NMR, UV, MS, HPLC, UPLC, and HSCCC, have been widely used for the characterization and quantification of IGs. Numerous lines of findings have elucidated the pharmacological activities of IGs. These studies have focused on multiple properties of IGs, such as their antioxidant, anti-inflammatory, or anticancer capacities. In recent years, IGs have attracted more attention due to their health-promoting effects on diabetes, obesity, liver injury, and thrombosis. Furthermore, the sugar residues of IGs make them more bioaccessible than aglycones. Meanwhile, IGs maintain higher plasma concentrations and longer average residence time in the blood than aglycons. This indicates that IGs are potent phytonutrients with potential health-promoting effects.

Growing evidence based on observational and clinical studies suggests that a plant-based diet based on fruits, vegetables, and whole grains has a significant effect on preventing various chronic diseases, including cancer, diabetes, and obesity [5]. IG traces have been identified in *Hippophae rhamnoides*, *Opuntia ficus-indica*, *Vaccinium corymbosum*, *Vaccinium myrtillus*, *Brassica juncea*, rice, onions, *Ginkgo biloba*, pollen Typhae, Microctis folium, *Sambucus nigra*, and *Calendula officinalis*, among their dietary and medicinal components [8,9,10]. People are more comfortable consuming phytochemicals and nutrients in their daily diets, such as fruit, vegetable juice, and tea [250]. They make vegetables and fruits into salads, blend them in juices, and process them into by-products. *Hippophae rhamnoides* could be served in pure juices, wine, and health supplements [251]. Meanwhile, *Opuntia ficus-indica* is used in many forms, including in food, feed, health, and nutrition, and is also used in formulated products, including teas, jams, and juices [252]. Additionally, IGs could be ingested from these plants. The extensive studies herein provide a sufficiently solid basis to discuss the health claims and health-promoting biological activities of IGs in humans. However, the clinical pharmacological effects of Igs still require further study so that their protective effects can be fully exploited in medical or pharmaceutical settings. The pharmacological mechanism of IGs also needs to be further elucidated to provide a material basis for their clinical investigation and application.

## Figures and Tables

**Table 1:** Isorhamnetin glycoside (IG) compounds (**1–49**). According to the number of sugar groups, IGs are divided into monoglycosides (**1–9**), diglycosides (**10–34**), triglycosides (**35–48**), and tetraglycosides (**49**).

No.	Name	Trivial Name	Source	Ref.
Monoglycosides				
1	Isorhamnetin-7-*O*-β-d-glucoside	Brassicin	*Centaurea cyanus* *Centaurea kotschyi* var. *kotschyi* *Cnicus wallichi* *Russowia Sogdiana* *Tagetes lucida* (Asteraceae) *Sedum sarmentosum* Bunge *Nitraria tangutorum* Bolor	[29] [30] [31] [32] [33] [34] [22]
2	Isorhamnetin-7-*O*-α-l-rhamnoside		*Carduncellus eriocephalus* *Nitraria tangutorum* Bolor *Atriplex centralasiatica* *Laportea bulbifera* Wedd. *V. galamensis* ssp. *galamensis var. petitiana* (A. Rich) M. Gilbert *Raphanus raphanistrum* L. *Caragana intermedia*	[35] [22] [36] [21] [37] [38] [39]
3	Isorhamnetin-3-*O*-α-l-rhamnoside		*Laportea bulbifera* Wedd.	[21]
4	Isorhamnentin-3-*O*-β-d-glucoside		*Astragalus centralpinus* *Solidago canadensis* L. *Hippophae rhamnoids* *Sambucus nigra* L. *Calendula officinalis*	[40] [28] [20] [41] [42]
5	Isorhamnetin-3-*O*-β-d-glucuronide		*Arnica montana* *Persicaria thunbergii* *Senecio giganteus* *Polygonum aviculare* L. *Senecio argunensis* Turcz.	[43] [44] [45] [46] [47]
6	Isorhamnetin-3-*O*-β-d-(2-acetyl-glucuronide)		*Polygonum aviculare* L.	[46]
7	Isorhamnetin-3-*O*-β-d (6-acetyl-glucoside)		*Solidago canadensis* L.	[28]
8	Isorhamnetin-3-*O*-β-d-galactoside		*Senecio argunensis* Turcz.	[47]
9	Isorhamnetin-4′-*O*-β-d glucoside		*Allium cepa* L.	[23]
Diglycosides				
10	Isorhamnetin-3-*O*-[2‴-*O*-acetyl−β-d-xyloside-(1→6)-β-d-glucoside]		*Gymnocarpos decander*	[27]
11	Isorhamnetin-3-*O*-[2‴,3‴-*O*-isopropylidene-α-l-rhamnoside]—(1→6)-β-d-glucoside		*Tetraena aegyptia*	[48]
12	Isorhamnetin-7-*O*-α-l-rhamnoside-(1→2)-β-d-glucoside	Isorhamnetin-7-*O*-β-neohesperidoside	*Cleome droserifolia*	[12]
13	Isorhamnetin-7-*O*-β-d-glucoside-(1→6)-β-d-glucoside	Astragaloside or Isorhamnetin-7-*O*-gentiobioside	*Astragalus altaicus*	[49]
14	Isorhamnetin-3-*O*-β-(4‴-p-coumaroyl-α-rhamnosy]—(1→6)-galactoside)		*Aerva javanica*	[50]
15	Isorhamnetin-3-*O*-α-l-rhamnoside-(1→2)-β-d-glucoside	Isorhamnetin-3-*O*-β-neohesperidoside	*Hippophae rhamnoids* *Typha augustifolia* L. *Calendula officinalis*	[20] [51] [42]
16	Isorhamnetin-3-*O*-β-d-xylosidel-(1→2)-β-d-galactoside		*Prunus padus* L.	[52]
17	Isorhamnetin-3,4′-*O*-β-d-diglucoside		*Allium ascalonicum* *Lepidium apetalum* willd	[24] [53]
18	Isorhamnetin-3,7-*O*-β-d-diglucoside		*Sedum sarmentosum* Bunge *Carduncellus eriocephalus*	[34] [35]
19	Isorhamnetin-3,7-*O*-α-l-dirhamnoside		*Laportea bulbifera* Wedd.	[21]
20	Isorhamnetin-3-*O*-β-d-glucoside-7-*O*-α-l-rhamnoside	Brassidine	*Sinapis arvensis* *Atriplex centralasiatica* *Hippophae rhamnoids*	[54] [36] [20]
21	Isorhamnetin-3-*O*-β-d-glucoside-4′-*O*-β-d-xyloside		*Diplotaxis harra* (Forssk.) Boiss	[26]
22	Isorhamnetin-3-*O*-α-l-rhamnoside-(1→6)-β-d-galactoside	Isorhamnetin-3-*O*-robinobioside	*Nitraria retusa*	[55]
23	Isorhamnetin-3-*O*-α-rhamnoside-(1→2)-rhamnoside		*Laportea bulbifera* Wedd.	[21]
24	Isorhamnetin-3-*O*-α-l-rhamnoside-(1→6)-β-d-glucoside	Narcissin Isorhamnetin-3-*O*-rutinoside	*V. galamensis* ssp. *galamensis var.* petitiana (A. Rich) M. Gilbert *opuntia ficus-indica* *Hippophae rhamnoids* *Ginkgo biloba* *Sambucus nigra* L. *Calendula officinalis*	[37] [18] [20] [9,56] [41] [42]
25	Isorhamnetin-3-*O*-β-d-apioide (1→2)-β-d-galactoside		*V. galamensis* ssp. *galamensis var. petitiana* (A. Rich) M. Gilbert	[37]
26	Isorhamnetin-3-*O*-α-l-arabinoside-7-*O*-β-d-glucoside		*Callianthemum taipaicum* *Narcissus pseudonarcissus*	[57] [58]
27	Isorhamnetin-3-*O*-β-d- (6‴-p-coumaroyl-α-glucoside-(1→2)-rhamnoside)		*Ginkgo biloba*	[56]
28	Isorhamnetin-3-*O*-β-d-glucoside-(1→2)-α-l-rhamnoside		*Ginkgo biloba*	[56]
29	Isorhamnetin-3-*O*-[2‴-*O*-acetyl−α-l-arabinoside-(1→6)-β-d-galactoside]		*Trillium tschonoskii* Maxim. *Trillium apetalon* Makino. and *T. kamtschaticum* Pallas.	[59] [60]
30	Isorhamnetin-3-*O*−α-l-arabinoside-(1→6)-β-d-galactoside		*Trillium apetalon* Makino. and *T. kamtschaticum* Pallas.	[60]
31	Isorhamnetin-3-*O*-α-(4″-acetyl-rhamnoside)-7-*O*-α-rhamnoside		*Cleome droserifolia*	[12]
32	Isorhamnetin-3-*O*-β-d-glucoside-7-*O*-α-l-arabinoside		*Eschscholtzia* mexicana Greene	[61]
33	Isorhamnetin-3-*O*-α-l-rhamnoside(1→2)]-β-d-galactoside		*Glycine max* (L.) Merr.	[62]
34	Isorhamnetin-3-*O*-β-glucoside-7-*O*-α-(3″′-isovaleryl)-rhamnoside		*Lepidium apetalum*	[53]
Triglycosides				
35	Isorhamnetin-3-*O*-α-l-rhamnoside-(1→6)-β-d-glucoside-4′-*O*-β-d-glucoside	Isorhamnetin-3-rutinoside-4′-glucoside	*Mercurialis annua*	[26]
36	Isorhamnetin-3-*O*-(2^G^-β-d-apiofuranosyl) [2‴-*O*-acetyl−β-d-xyloside-(1→6)-β-d-glucoside]		*Gymnocarpos decander*	[27]
37	Isorhamnetin-3-*O*-(2″,6″-*O*-α-l-dirhamnoside)-β-d-galactoside		*Alangium premnifolium* *Lysimachia fortunei*	[63] [64]
38	Isorhamnetin-3-*O*-(4^Rham^-β-d-galactosyl)-α-l-rhamnoside-(1→6)-β-d-galactoside]	Isorhamnetin-3-*O*-4^Rham^-galactosyl-robinobioside	*Nitraria retusa*	[55,65]
39	Isorhamnetin-3-*O*-α-l-rhamnoside-(1→2)-β-d-galactoside-7-*O*-β-d-glucoside		*Blackstonia perfoliata*	[66]
40	Isorhamnetin-3-*O*-α-l-rhamnoside-(1→6)-β-d-glucoside-7-*O*-α-l-rhamnoside	Isorhamnetin-3-rutinoside-7-rhamnoside	*Cassia italica* *Hippophae rhamnoides*	[67] [68]
41	Isorhamnetin-3-*O*-β-glucoside-(1→2)-β-d-glucoside-7-β-d-glucoside	Brassicoside or Isorhamnetin-3-*O*-sophoroside-7-*O*-β-d-glucoside	*Brassica napus*	[54]
42	Isorhamnetin-3-*O*-β-d-xyloside-(1→3^Rham^)-α-l-rhamnoside-(1→6)-β-d-galactoside	Isorhamnetin 3-xylosyl-robinobioside	*Nitraria retusa*	[55]
43	Isorhamnetin-3-*O*-β-glucoside-(1→2)-β-d-glucoside-7-*O*-α-l-rhamnoside	Isorhamnetin-3-*O*-sophoroside-7-*O*-rhamnoside	*Hippophae rhamnoids*	[20]
44	Isorhamnetin-3-*O*-[(6-*O*-E-sinapoyl)-β-d-glucoside-(1 → 2)]-β-d-glucoside-7-*O*-α-l-rhamnoside		*Hippophae rhamnoids*	[20]
45	Isorhamnetin-3-*O*-(2^G^-α-l-rhamnoside)-α-l-rhamnoside-(1→6)-β-d-glucoside	Typhaneoside	*Typha augustifolia* L. *Calendula officinalis*	[51] [42]
46	Isorhamnetin-3-*O*-(2^G^-β-d-glucoside)-α-l-rhamnoside-(1→6)-β-d-glucoside		*Boldo Folium*	[69]
47	Isorhammetin-3-*O*-α-l-rhamnoside-(1→6)-β-d-glucoside-7-*O*-β-d-glucoside	Isorhammetin-3-rutinoside-7-glucoside	*Hippophae rhamnoids* *Mercurialis annua*	[20] [26]
48	Isorhamnetin-3-*O*-β-d-glucoside-7-*O*-β-d-glucoside-(1→6)-β-d-glucoside	Isorhamnetin-3-*O*-glucoside-7-*O*-gentiobioside	*Lepidium apetalum* willd	[53]
Tetraglycosides				
49	Isorhamnetin-3-*O*-[2^G^-α-l-rhamnoside-(1→6)-β-d-glucoside]-α-l-rhamnoside-(1→6)-β-d-glucoside		*Boldo Folium*	[69]

**Table 3:** Selected examples of marketed *Opuntia ficus-indica* products.

Product Type	Ingredients	Brand	Country
Tender nopalitos	Cladode	La Costena	Mexico
Sauce	Cladode, fruit	Marie Sharp’s, Navajo Mike’s	Belize, United States
Beer	Whole plant	Michelob Ultra	United States
Juice	Fruit	Dynamic Health, Maxx Herb	United States
Drink	Cladode	Yunseonae Cactus, San Pellegrino	Korea, Italian
Cocktail syrup	Fruit	The Prickly Pear Pantry	United States
Water	Fruit, whole plant	Pricklee, True Nopal	United States
Tea	Fruit	Snapple	United States
Tea bags	Cladode and fruit, fruit	Only Natural, Loyd	United States, Poland
Sugar	Cladode, fruit	HealthForce SuperFoods, Arizona Gifts	United States
Capsules	Cladode	Swanson, Solaray, Natural Home Cures, Tadin, Carlyle	United States
Tablets	Whole plant	Planetary Herbals	United States
Pills	Whole plant	Flyby	United States
Meal	Seed	Nuestra NS Salud	United States
Powder	Cladode	BareOrganics	United States
Liquid supplements	Fruit	Nochtli SuperiorFruit	United States
Drops	Whole plant	Natural Home Cures	United States
Campanelle pasta	Cladode	Merkin Vineyards	United States

**Table 4:** Selected examples of marketed *Hippophae rhamnoides* products.

Product	Ingredients	Brand	Country
Juice	Flesh, juice, skin, pulp, seed oil	Genesis Today, Dynamic Health, Tongrentang, Vitba	United States, United States, China, Russia
Oil	Seed, berry	SeabuckWonders, SIBU, PipingRock, Swanson	United States, United States, United States, United States
Pure	Berry	SIBU	United States
Powder	Berry	LOOV	Estonia
Tea	Leave, berry	OBH, Far East echipam, Apotheke, Xiuzheng, Wanmei	Lithuania, Korea, Germany, China, China
Capsules	Seed, berry	Terezia	Czech Republic

## Data Availability

Not applicable.

## Abbreviations

5-HMF	5-hydroxymethylfurfural
ABTS	2,2′-azino-bis(3-ethylbenzothiazoline-6-sulfonate)
ADH	alcohol dehydrogenase
AGEs	advanced glycation end products
ALDH	aldehyde dehydrogenase
ALT	alanine aminotransferase
Ara	l-arabinose
AST	aspartate aminotransferase
ATM	ataxia telangiectasia mutated gene
ATR	ATM and Rad3-related gene
BB	bee read
CCl_4_	carbon tetrachloride
Chk1	checkpoint kinase1
Chk2	checkpoint kinase2
CAA	cellular antioxidant activity assay
COX-2	cyclooxygenase-2
CUPRAC	CUPric reducing antioxidant capacity
DAD	diode array detection
DPPH	2,2-diphenyl-1-picrylhydrazil
DPP-IV	dipeptidyl peptidase-IV
DW	dry weight
ERK	extracellular regulated kinases
ESI	electrospray ionization
FRAP	ferric reducing antioxidant power
Gal	d-galactose
Glc	d-glucose
Glccur	d-glucuronic
HFD	high-fat diet
HHP	high hydrostatic pressure
HMGB1	high-mobility-group protein 1
HO-1	heme oxygenase-1
HPLC	high-performance liquid chromatography
HSCCC	high-speed counter-current chromatography
HSCs	hepatic stellate cells
IGs	isorhamnetin glycosides
IL-6	interleukin-6
IL-1β	interleukin-1β
iNOS	inducible nitric oxide synthase
IR	infrared spectroscopy
JNK	c-Jun *N*-terminal kinase
LPS	lipopolysaccharide
MAPK	mitogen-activated protein kinase
MDA	malondialdehyde
MEOS	microsomal ethanol oxidizing system
MS	mass spectrometry
NF-κB	nuclear factor kappa-B
NMR	nuclear magnetic resonance
Nrf2	nuclear factor E2-related factor 2
OFI-E	*opuntia ficus-indica* extract
ONOO(-)	peroxynitrite
ORAC	oxygen radical absorbance capacity
PAI-1	plasminogen activator inhibitor type 1
PBMC	human peripheral blood mononuclear cells
PSC	peroxyl radical-scavenging capacity
PTP1B	protein tyrosine phosphatase 1B
Rha	l-rhamnose
ROS	reactive oxygen species
Xyl	D-xylose
TGF-β	transforming growth factor-β
TLC	thin-layer chromatography
TNF-α	tumor necrosis factor
tPA	tissue-type plasminogen activator
UPLC	ultra-performance liquid chromatography
UV	ultraviolet radiation
α-SMA	alpha-smooth muscle actin
